# Dynamics of Blood Count after Rheohemapheresis in Age-Related Macular Degeneration: Possible Association with Clinical Changes

**DOI:** 10.1155/2014/858219

**Published:** 2014-03-06

**Authors:** Milan Košťál, Milan Bláha, Eva Rencová, Miriam Lánská, Pavel Rozsíval, Vera Kratochvilová, Hana Langrová

**Affiliations:** ^1^IVth Department of Internal Medicine-Hematology, Faculty Hospital, Faculty of Medicine in Hradec Králové-Charles University in Prague, Sokolska Street 581, 500 05 Hradec Králové, Czech Republic; ^2^Department of Ophthalmology, Faculty Hospital, Faculty of Medicine in Hradec Králové-Charles University in Prague, Sokolska Street 581, 500 05 Hradec Králové, Czech Republic

## Abstract

*Background*. Rheohemapheresis (RHF) is a method that can stop the activity of the dry form of age-related macular degeneration (AMD). The pathophysiologic mechanisms are not well understood, and the effects of the RHF procedures extend beyond the time of the individual procedures. *Patients and Methods*. We present the data for 46 patients with AMD treated with a series of 8 rheohemapheretic procedures. Blood count parameters were measured before the first and the last procedures. The clinical effect was judged by changes in the drusenoid pigment epithelium detachment (DPED) area before and after the rheopheretic sessions. *Results*. Rheopheresis caused a decrease in hemoglobin (*P* < 0.001), a decrease in leukocytes (*P* < 0.034), and an increase in platelets (*P* < 0.005). We found a negative correlation between the amount of platelets and their volume (*P* < 0.001, Pearson correlation coefficient: −0.509). We identified the platelet/MPV ratio as a good predictor of the clinical outcome. Patients with a platelet/MPV ratio greater than 21.5 (before the last rheopheresis) had a significantly better outcome (*P* = 0.003, sensitivity of 76.9% and specificity of 80%). *Conclusion*. Several basic blood count parameters after RHF can be concluded to significantly change, with some of those changes correlating with the clinical results (reduction of the DPED area).

## 1. Introduction

Age-related macular degeneration (AMD) is the main cause of legal blindness among the elderly [1–4]. AMD usually begins as the dry form of the disease. Arresting the progression of the disease with effective treatment at this stage, when the vision is not markedly damaged, would be desirable. However, there is no proven therapy for advanced dry macular degeneration. The rheological aspects of the disease may be the key for treatment. One such therapy was described by Klingel et al. [4–7]. The methodology and clinical possibilities of rheological therapy (e.g., rheopheresis, rheohemapheresis, and hemorheopheresis) and the importance of microvascular flow were verified [4, 6]. The aim of the therapy is to stop the progression of the disease. In several cases, we observed certain improvements, such as the disappearance of the drusen or a reduction of thedrusenoid retinal pigment epithelium detachment (DPED) area [8–10].

During the course of AMD, periods of both long-term stabilization and sudden turns for the worse may occur. Therefore, recognizing the disease activity in time and stopping its progress by RHF are necessary. During our long-term research of AMD and other microcirculation disorders, we have studied markers of clinical activity and the possibilities of improving the clinical state after extracorporeal elimination. We have found changes in markers of apoptosis, several cytokines, interleukins, or endoglin [11]. Obtaining these markers is technically difficult and relatively expensive, and the results are not immediately available. However, we identified several interesting changes in the complete blood count that could serve as easily available markers of the therapy effectiveness. Determining the changes in the blood counts and understanding their relationship with clinical improvement (the DPED resorption) are the objectives of this study.

## 2. Materials and Methods

### 2.1. Patients

The 46 patients included in this study, 17 men and 29 women, suffered from the dry form of age-related macular degeneration. The mean age of the patients was 68.9 (±7.2) years, and their age range was 53–86 years. The patients had to have a diagnosis of AMD in both eyes, with dry AMD in one or both eyes, confirmed by fluorescein angiography and fundus photography. The controls were recruited from healthy blood donors: 20 men and 50 women. The mean age of the controls was 41 ± 10.3 years, and their age range was 19–61 years.

### 2.2. Rheohemapheresis

Our modification of the cascade method (named rheohemapheresis) was used for the therapy of our patients. After plasma separation (blood-cell separator, Cobe Spectra, Denver, USA), the separated plasma was pumped through a rheofilter (Evaflux 4A, Kuraray, Osaka, Japan). The size of the filter pores enabled a considerable amount of high molecular weight (HMW), rheologically active substances (such as LDL-cholesterol, lipoprotein(a), fibrinogen, *α*2-macroglobulin, and immunoglobulins, especially IgM) to be captured. The amount of washed plasma was calculated by the computer of the Cobe Spectra separator. In each therapeutic series, the amount washed was 1 body volume during the first procedure (for safety reasons) and 1.5 body volumes in all the following procedures. The plasma flow was continual, and anticoagulation was performed with a basic bolus of heparin and then continually with an ACD-A (Baxter, USA) solution. Every patient was treated with a series of 8 RHF procedures over 10 weeks (twice, every other day, in the first week, followed by 2 weeks rest period and repeated again 4 times). The procedures used have been previously described elsewhere [8–10].

### 2.3. Laboratory Examinations

Blood samples were collected before and after the first and the eighth (the last) sessions. The blood samples were measured at accredited laboratories (Faculty Hospital, Hradec Kralove, CZ) with standard methods using the Sysmex XE-2100 device (Sysmex Corporation, Kobe, Japan). Determination of the complete blood count was performed using 100 *μ*L of blood, and the response time was approximately 15 minutes after the laboratory received the sample.

### 2.4. DPED Evaluation

Optical coherence tomography (OCT) was performed using a Stratus OCT (OCT 3 Stratus, Zeiss, Jena, Germany) before and 2.5 years after the therapy. DPED was found in 36 eyes. The subsequent fundus photography was performed to evaluate the DPED area. The Visupac method (Visupac System, Zeiss, Jena, Germany, which measures the DPED area in mm^2^) outlined the DPED area, and a copy of this area was transferred to the studied picture with special software.

### 2.5. Statistics

Univariate comparisons of continuous variables within groups were performed with the paired* t*-test or the nonparametric Wilcoxon rank sum test in cases of nonnormally distributed variables. For independent groups, the unpaired* t*-test or nonparametric Mann-Whitney rank sum test was used. The association of platelet number and its volume before and after the rheopheretic series was tested using the Pearson product moment correlation. The statistical analyses were performed with SigmaPlot for Windows, version 11. The results are presented as the means (±SD). The platelet (10^9^/L)/MPV (fL) ratio for the prediction of clinical outcome was tested with ROC (receiver operating characteristic) curve analysis. After generating the ROC curves, we evaluated the criterion value, sensitivity, specificity, and area under the curve (AUC) as a marker of the test quality (AUC: 1–0.97, excellent; 0.97–0.92, very good; 0.92–0.75, good; 0.75–0.50, applicable test). The ROC analysis was performed in MedCalc 12.4 for Windows.

## 3. Results

**Figure 1:**
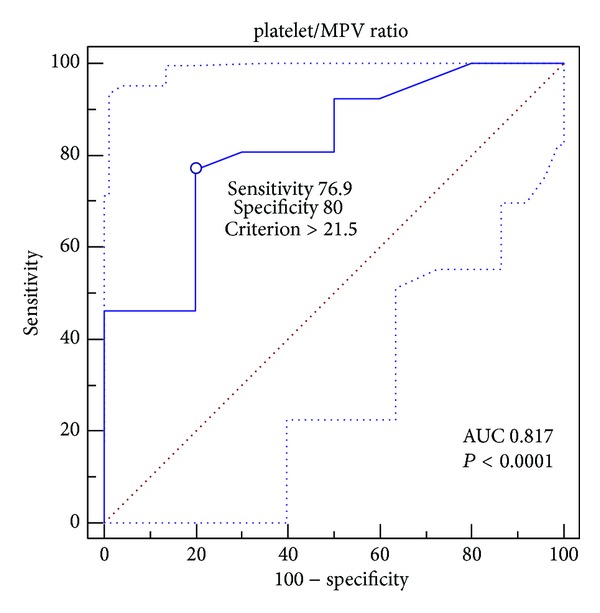
ROC curve for determination of method sensitivity and specificity at cut-off value of platelet/MPV ratio of 21.5.

The blood count, fibrinogen, and cholesterol changes before the first and last sessions are shown in Table 1. The hemoglobin level dropped during the procedures (*P* < 0.001) until 10 weeks after the therapy initiation. The leukocyte levels were raised after the first procedure but were reduced before the last session (*P* < 0.034). The comparison of the leukocyte levels before the start of apheresis revealed that the number of leukocytes was elevated in the patients compared with the healthy controls (*P* = 0.001) (Table 2). The number of platelets increased during the procedures (*P* < 0.005). When searching for an association among the changes in selected parameters, we found a negative association between the difference in the platelet number and its volume (*P* < 0.001, Pearson correlation coefficient: −0.509). The platelet numbers were reduced with each procedure (median 226 versus 216 before and after each procedure) (*P* < 0.001). However, during the long-term course, the platelet quantity was increased (224 before the first procedure versus 235 before the last procedure) (*P* < 0.001). To evaluate the clinical effect (change in the size of the DPED area before and after the treatment), we used the platelet/MPV ratio. Using ROC analysis for the prediction of a favorable prognosis, we determined that the cutoff of 21.5 (*P* < 0.001) had a sensitivity of 76.9%, a specificity of 80.0%, and an AUC of 0.817 (good quality) (Figure 1). Positive predictive value is 90.9% and negative predictive value is 57.1%. Patients with a platelet/MPV ratio greater than 21.5 before the last RHF had a significantly better outcome (*P* = 0.003, mean regression in the DPED area of 3.35 mm^2^ [±2.88] versus 0.344 mm^2^ [±2.63]).

## 4. Discussion

RHF is used for the treatment of sudden hearing loss, age-related macular degeneration, peripheral arterial disease, and several other microcirculatory diseases [6, 7, 12, 13]. Data supporting the efficacy of this procedure have been published, but detailed pathophysiological explanations of the positive clinical effects are not clear or are missing. However, several possible mechanisms have been proposed. In our previous study, we demonstrated that there is a reduction in the blood viscosity in AMD [9]. After RHF, a decrease in the macromolecule concentrations (mainly fibrinogen) occurred [14], whereas erythrocyte deformability improved [13]. Together, both led to improved blood supply in the microcirculation. Improved microcirculation could arrest the disease progression and lead to a substantial improvement in the visual function in people suffering from AMD [15, 16]. Several other factors may play important roles; for example, endothelial progenitor cells (EPC) derived from the bone marrow might mediate a long-term effect. Therapy with statins, erythropoietin, or estrogens demonstrated an increase in the circulating EPC [17]. Therapeutic apheresis was capable of mobilizing such cells [18]. Removing cytokines, inflammatory mediators, or yet-unknown plasma components might be another method to induce the long-term clinical effects of RHF. Thus, there appears to be a “pleiotropic effect” similar to that described in LDL apheresis [17].

According to the recent literature concerning AMD, significant changes in the complement system have been observed, mainly in activation of several complement components [19]. The activity changes are time-dependent. Currently, we hypothesize that RHF favorably influences several clinical changes, including vision [10], and favorably influences the pathways of complement activation and H protein regulation. Indeed, the first results showed the decreasing activity of several complement components after rheohemapheresis (data unpublished).

Our study showed immediate changes in the blood count parameters after a single apheresis treatment, and long-term changes were induced over the entire rheopheretic therapy series. The decrease in the hemoglobin level was most likely the result of a small blood loss in the device (10–30 mL, according to the manufacturer). This change is logical and was observed during the entire RHF course. This small amount of blood loss can be presumed to be replaced with increased production during the intervals between the procedures. Any differences between the hemoglobin values in the controls and patients before the therapy were not observed.

In the leukocytes, in contrast, the admission value was higher (not clinically dangerous) in patients than in healthy controls (*P* < 0.001). We did not try to search for age and sex matched healthy controls because neither of these parameters has a significant impact on leukocytes level [20]. Higher levels of leukocytes may be a negative prognostic factor of the disease, as in several other diseases. Twig et al. described a higher risk of coronary artery disease in patients with leukocytes in the upper 2 quintiles [21]. Similarly, other authors described an increased level of leukocytes as a risk factor for peripheral artery disease [22], heart failure [23], or ischemic cardiovascular disease [24]. No data from the literature have been found regarding increased leukocyte values or their importance in AMD. During the course of a single apheresis treatment, the leukocyte level increased due to the leukocyte activation caused by the contact with the microenvironment in the separator tubes or rheopheretic filter. During the 7 cycles, the leukocyte value decreased significantly, which likely corresponded with the clinical benefits of rheohemapheresis (together with the decreases in the LDL-cholesterol and fibrinogen levels).

The number of thrombocytes (and their MPV) prior to the rheopheresis in the patients was the same as in the controls. In a single procedure, the number of thrombocytes (and their MPV) is well known to decrease due to their losses in the separator tubes or the rheopheretic filter (herein, this loss is likely larger, with more active platelets being caught). However, during rheopheresis, their numbers increase. Additionally, Macher et al. described an elevation of thrombocytes in donors 14–42 days following multicomponent apheresis [25]. The generation of thrombocytes is regulated by thrombopoietin, and thrombopoietin is increasingly washed out with the decrease in platelet number [26]. Other possible factors influencing platelet generation are granulocyte-colony stimulating factors, interleukins, and nitrogen oxides [27]. These agents are increasingly released in endothelial dysfunction, typically in diabetes, brain stroke, and cardiac ischemia [28]. The volume and shape of the platelets also increase during their activation. Larger platelets contain more granules, thromboxan A2, serotonin, and ATP and express more adhesive molecules (P-selectin, GpIIb/IIIa) [29]. Increased platelet volume has been described in patients with diabetes, coronary affection, obesity, hypercholesterolemia, ictus [30, 31], and other diseases. Therefore, we believed that also evaluating the MCV in patients with AMD would be useful.

In our group of patients, the platelet volume was negatively correlated with the platelet number. Brækkan et al. described such negative dependence in a sample of 25,923 healthy participants (Pearson correlation coefficient = −0.47, *P* < 0.001) [32]. An increased number of smaller platelets appeared to be produced during thrombopoiesis activation and vice versa. With regard to the demonstrated relationship between higher MPV and changes in microcirculation or in atherosclerotic complications [30, 31], the increased number of smaller thrombocytes in AMD may be considered to be prognostically more favorable.

The long-term efficacy of the dry AMD therapy with rheohemapheresis in our group of patients was demonstrated previously [8].

To determine the changes in the DPED area, we measured the area before therapy and then 2.5 years after the therapy had been completed (see our previous study [33]). The area decreased with time compared with the controls; the increased area in the controls was significant (*P* < 0.001). Our present-day study confirmed the positive relationship of the platelet count/MPV ratio with the change in the DPED area and that the clinical result of the RHF therapy was associated with clinical improvement (higher platelet/MPV ratio after RHF therapy = regression of the DPED area). This relationship is not causal (i.e., the higher platelet count and clinical improvement are apparently a consequence caused by the activity of other factors), but it may serve as an inexpensive and easily available prognostic biomarker with sufficient sensitivity and specificity. We can presume that this marker could be the guide for the intensity (additional cycles) of the treatment. To the best of our knowledge, there are no publications with similar results. Further research is needed with more patients to verify these observations.

## 5. Conclusion

The basic blood count parameters significantly changed during the rheohemapheresis treatments. Although these changes are not clinically significant, they may be highly significant for research. The changes occur not only after individual RHF procedures (see above) but also during long-term followup due to changes in the microenvironment. The thrombocyte count/MPV ratio at the end of the rheohemapheresis therapy may serve as a laboratory biomarker for patient prognosis.

## Figures and Tables

**Table 1:** Blood count before the first and the last procedures.

	Before the 1st	Before the 8th	*P*
Hemoglobin (g/L)	134.24 (22.24)	128.22 (14.0)	<0.001
Leukocytes (10^9^/L)	6.77 (1.92)	6.55 (1.41)	0.034
Platelets (10^6^/L)	224.46 (45.15)	235.54 (50.32)	0.005
MPV (fL)	10.63 (1.11)	10.58 (1.11)	0.548
Fibrinogen (g/L)	3.71 (0.61)	2.71 (0.67)	<0.001
LDL-cholesterol (mmol)	2.40 (0.90)	2.15 (0.75)	0.046

**Table 2:** Comparison of blood count in the patients and healthy controls before the therapy.

	Patients	Controls	*P*
Hemoglobin (g/L)	134.24 (22.24)	138.96 (1.36)	0.173
Leukocytes (10^9^/L)	6.77 (1.92)	5.94 (1.35)	0.001
Platelets (10^6^/L)	224.46 (45.15)	224.67 (56.31)	0.42
MPV (fL)	10.63 (1.11)	10.47 (0.78)	0.46
